# Evans syndrome during pembrolizumab therapy for upper urinary tract cancer

**DOI:** 10.1002/iju5.12609

**Published:** 2023-07-27

**Authors:** Shota Kakita, Tomohiro Matsuo, Masaharu Ohki, Ayaka Tsuchiyama, Takuji Yasuda, Hiromi Nakanishi, Kensuke Mitsunari, Kojiro Ohba, Ryoichi Imamura

**Affiliations:** ^1^ Department of Urology Nagasaki University Graduate School of Biomedical Sciences Nagasaki Japan

**Keywords:** autoimmune hemolytic anemia, Evans syndrome, immune checkpoint inhibitor, immune thrombocytopenic purpura, immune‐related adverse event

## Abstract

**Introduction:**

Immune checkpoint inhibitors are available for the treatment of advanced urothelial carcinoma; however, serious adverse events occasionally occur. Here, we report a rare case of Evans syndrome attributed to the use of an immune checkpoint inhibitor.

**Case presentation:**

A 56‐year‐old man was diagnosed with left renal pelvic cancer and underwent left laparoscopic radical nephroureterectomy. Eight months postoperatively, computed tomography revealed para‐aortic lymph node metastasis. Despite receiving chemotherapy, the disease progressed, and pembrolizumab was initiated. After 26 months of pembrolizumab treatment, the patient developed fever and anemia. Hematologic examination confirmed the diagnosis of Evans syndrome. He was treated with blood transfusions and corticosteroids, and gradual symptom improvement was observed.

**Conclusion:**

This report highlights the potential risk of Evans syndrome associated with immune checkpoint inhibitor treatment. Clinicians should be aware of this possibility and consider early intervention with corticosteroids.

**Abbreviations & Acronyms**
AIHA: autoimmune hemolytic anemia
ALP: alkaline phosphatase
ALT: alanine aminotransferase
APTT: activated partial thromboplastin time
AST: aspartate aminotransferase
CT: computed tomography
ES: Evans syndrome
GTP: glutamyl transpeptidase
ICI: immune checkpoint inhibitor
irAE: immune‐related adverse event
ITP: immune thrombocytopenic purpura
IVIg: intravenous immunoglobulin
LDH: lactate dehydrogenase
Pem: pembrolizumab
PT: prothrombin time
PT‐INR: prothrombin time‐international normalized ratio
RBC: red blood cell
UC: urothelial carcinoma

**Keynote message**

Evans syndrome is a rare adverse event associated with immune checkpoint inhibitor treatment. If a patient presents with severe anemia and thrombocytopenia during immune checkpoint inhibitor treatment, Evans syndrome should be considered in the differential diagnosis for further investigation.

## Introduction

The ICI, Pem, has been indicated for treatment of unresectable UC that has progressed after chemotherapy. Although this treatment has shown improved outcomes for UC, some patients may experience irAEs.

Here, we report a rare case of ES complicated by AIHA and ITP as irAEs in a patient receiving Pem for upper urinary tract UC.

## Case presentation

A 56‐year‐old man with gross hematuria underwent left laparoscopic radical nephroureterectomy. The pathological diagnosis was UTUC (pT2N0M0). Eight months postoperatively, CT revealed metastasis to the para‐aortic lymph nodes; thus, gemcitabine and carboplatin chemotherapy was initiated. However, after two cycles of chemotherapy, further metastatic lymph node enlargement was observed, indicating progressive disease.

After 26 months (33 cycles) of Pem treatment, the patient developed fever and general fatigue. Physical examination revealed pale eyelid conjunctiva and yellowish ocular conjunctiva. Additionally, the patient experienced mild subcutaneous bleeding in the lower extremities.

**Fig. 1:**
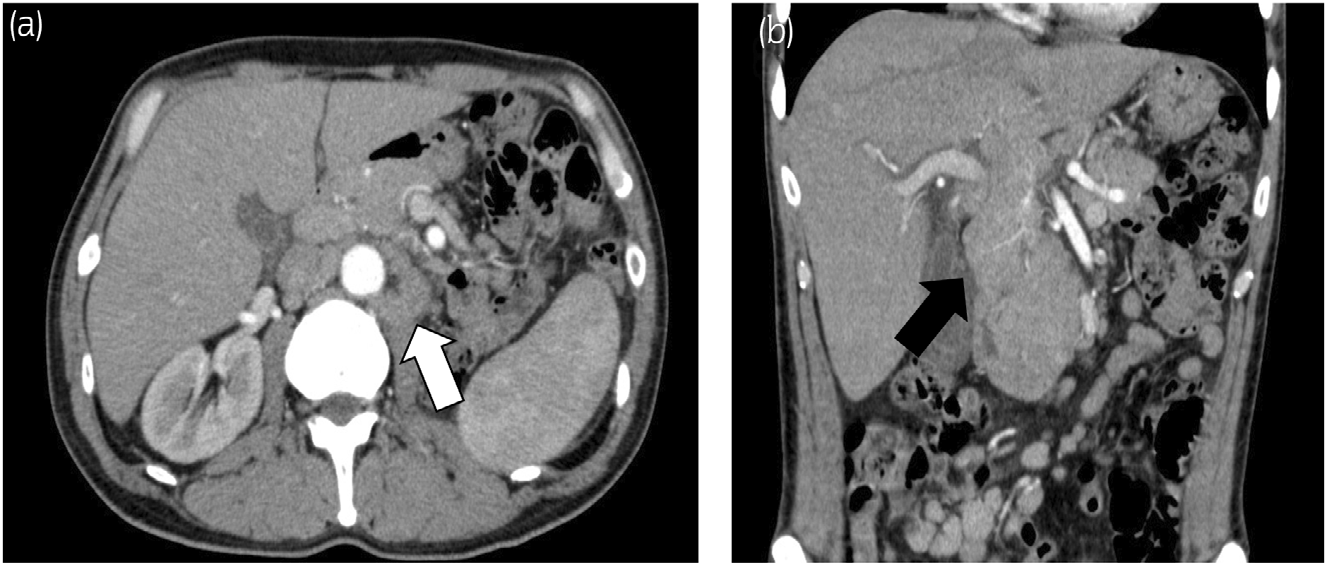
Abdominal computed tomography findings. An enlarged para‐aortic lymph node (white arrow) is visible; however, it did not significantly differ from before Pem treatment (a). The hilar and mesenteric lymph nodes (black arrow) are prominently enlarged (b).

Blood tests revealed severe anemia, low platelet count, and elevated LDH level. Elevated liver enzymes and bilirubin levels indicated hemolysis or worsening liver function (Table 1). Contrast‐enhanced CT revealed hepatosplenomegaly and enlarged lymph nodes in the mesentery and hepatic hilum but no evidence of bleeding or infection (Fig. 1). A blood transfusion was determined to be necessary. Further examination revealed positive results for irregular antibodies and direct and indirect Coombs tests (Table 1). Bone marrow puncture revealed no malignant findings. No evidence of hemorrhage, disseminated intravascular coagulation, or primary hepatobiliary disease was found, and other hematologic diseases, such as malignant lymphoma, were ruled out. Finally, based on the diagnostic criteria set forth by the Ministry of Health, Labor, and Welfare, we diagnosed the patient with ES as he met the criteria for both AIHA and ITP.^1^, ^2^

**Table 1:** Results of laboratory investigations at baseline and the onset of ES.

	Baseline	At the onset of EV
Hematology		
White blood cell (×10^3^/μL)	5.5	8.9
Red blood cell (×10^6^/μL)	3.74	1.52
Reticulocyte (%)	—	3.75
Hemoglobin (g/dL)	10.5	4.7
Platelets (×10^3^/μL)	195.0	81.0
Biochemistry		
Total protein (g/dL)	7.9	8.1
Albumin (g/dL)	2.8	2.6
Total bilirubin (mg/dL)	0.5	2.7
Direct bilirubin (mg/dL)	—	1.7
Indirect bilirubin (mg/dL)	—	1.0
AST (U/L)	19	69
ALT (U/L)	20	113
Gamma‐GTP (U/L)	20	223
ALP (U/L)	70	518
LDH (U/L)	110	223
C‐reactive protein (mg/dL)	0.23	7.26
Blood urea nitrogen (mg/dL)	24	18
Creatinine (mg/dL)	1.38	1.21
Uric acid (mg/dL)	—	7.7
Sodium (mmol/L)	141	133
Potassium (mmol/L)	4.2	4.8
Chloride (mmol/L)	105	100
Coagulation		
PT (%)	—	81
PT‐INR	—	1.13
APTT (Sec)	—	31.2
D‐dimer (μg/mL)	—	0.7
Immunology		
Cold agglutination reaction	—	256
Direct Coombs test	—	Positive
Indirect Coombs test	—	Positive
Donath‐Landsteiner	—	Negative

**Fig. 2:**
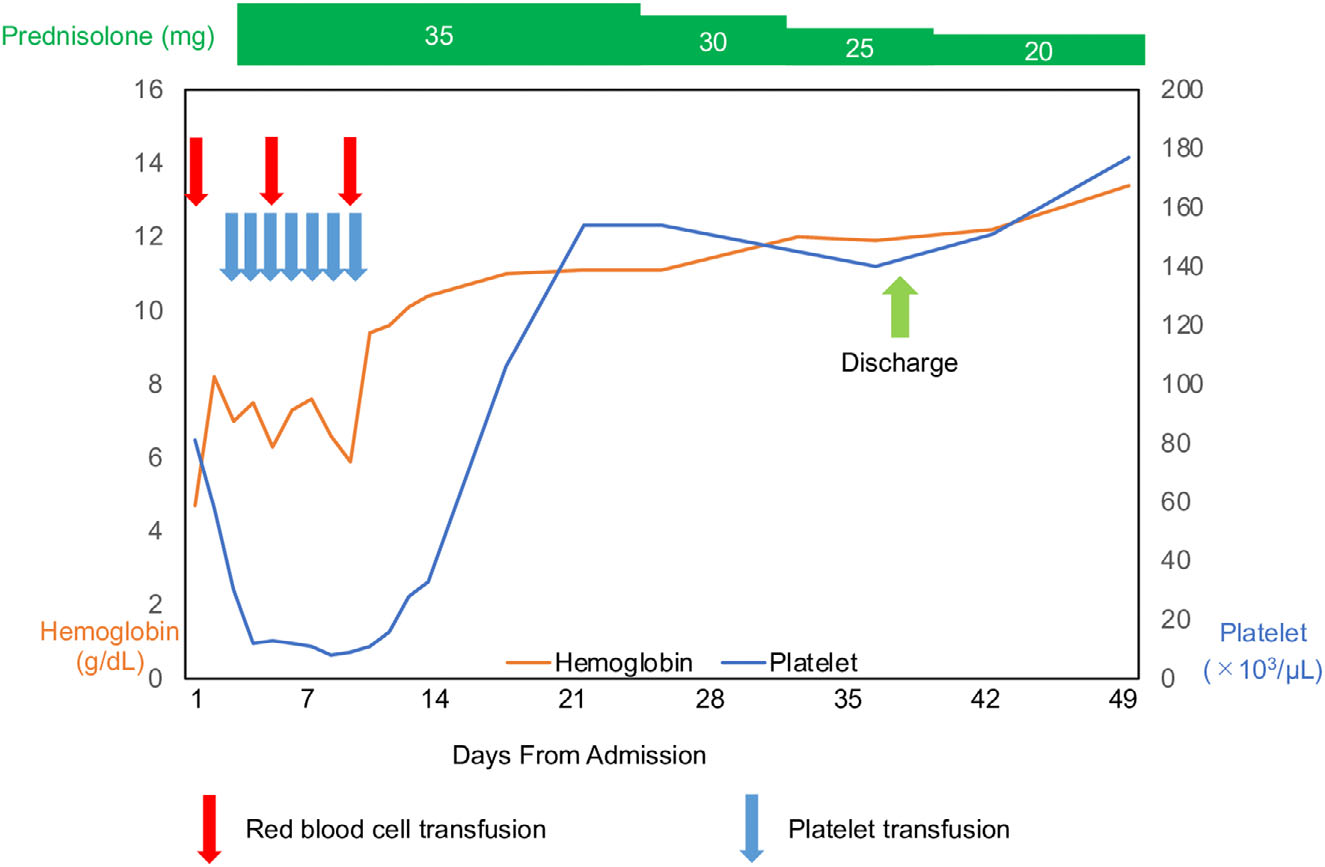
The clinical course after admission. Steroid therapy was remarkably effective; hemoglobin and platelet levels improved.

Upon admission, oral prednisolone (35 mg/day) administration was initiated. RBC and platelet transfusions were deemed necessary; however, both improved over time (Fig. 2). Following 34 days of oral prednisone treatment, the patient was discharged and has since maintained stable health with oral prednisone (5 mg/day).

## Discussion

The patient developed ES as an irAE during second‐line Pem therapy for metastatic UTUC following chemotherapy. The patient achieved remission after prompt diagnosis and oral steroid therapy.

ES was first described in 1951 as an autoimmune disease concomitant or secondary to AIHA or ITP.^3^ Approximately 50% of ES cases are classified as secondary ES, which is associated with a higher mortality rate than primary ES. The etiology of ES should be accurately determined at the time of diagnosis, as it may influence subsequent treatment strategies.^4^ Autoimmune diseases and lymphomas are the most common causes of secondary ES.^4^ Overall, 61% of the cases were reported to develop AIHA and ITP simultaneously, while 39% exhibit asynchronous onset. However, a clear trend regarding which disease manifested first was not established.^4^

As this patient experienced anemia; jaundice, with elevated reticulocyte, indirect bilirubin, and LDH; AIHA; and ITP with thrombocytopenia, drug‐induced secondary ES was strongly suggested. Additionally, considering the occurrence of anemia and thrombocytopenia in the patient, we concluded that it was a case of ES with almost simultaneous development of AIHA and ITP.

ICIs have been increasingly used as systemic therapy for advanced malignancies. While ICIs have demonstrated anticancer effects, their use is associated with the loss of self‐tolerance and the occurrence of irAEs.^5^

The incidence of hematologic toxicity due to immunotherapy has been reported to be as high as 1%.^6^ AIHA, specifically attributed to Pem, is considered rare, accounting for only 0.146% of all AEs caused by Pem.^7^ Nonetheless, approximately 30% of patients with AIHAs presenting as irAEs have new‐onset or complications of other hematologic diseases such as ITP.^8^ Leaf *et al*. reported 14 cases of AIHA caused by ICIs, four of which (28.6%) had additional hematological toxicity. The breakdown of the primary disease was melanoma in two out of these four cases (50%), non‐small cell lung cancer in one case (25%), and acute myeloid leukemia in one case (25%).^8^ Therefore, clinicians should excise caution and consider the possibility of ES when managing patients with hematologic abnormalities.

Corticosteroids, particularly prednisolone, are the primary therapy for ES, typically administered at a dose range of 1–2 mg/kg/day.^9^ To minimize the risk of steroid‐associated AEs, gradually tapering the steroid dose based on the patient's clinical presentation is crucial.^10^ When steroid treatment proves ineffective, second‐line options, such as rituximab, may be considered. However, limited data are available regarding its efficacy in patients with secondary ES.^10^, ^11^

In cases of pronounced thrombocytopenia, IVIg should be considered as a potential treatment option.^12^ IVIg may serve as a primary treatment in cases where steroid therapy is not indicated or not suitable. However, when combined with steroids, it may lead to a rapid increase in platelet count.^13^

Our patient was managed with a daily dose of prednisolone at 0.5 mg/kg (equivalent to 35 mg/day). The steroid dosage used in this study was relatively lower than that reported previously. This decision was based on the judgment of our consulting hematologist, who believed that high doses and continuous steroid administration carried a higher risk for of AEs in this case. The management of ES typically reserves RBC transfusions for patients experiencing symptoms or when the condition becomes life‐threatening. However, in our patient, an RBC transfusion was performed owing to a marked decrease in RBC count and mild hypotension observed at the initiation of steroid therapy. Aggressive platelet administration is not routine practice in patients with ES, as platelets have a short half‐life and achieving significant improvement in patient outcomes is challenging.^14^ However, in this case, the patient had severe thrombocytopenia, and platelet transfusions were administered after consultation with a hematologist.

A review by Tanios *et al*. demonstrated that the first report on Pem‐induced hematologic toxicity was published in 2014.^7^ Subsequently, as the indications for Pem have increased, including malignant melanoma,^15^, ^16^ non‐small cell lung cancer,^17^, ^18^ and chronic lymphocytic leukemia,^19^ there have been increased in reports of hemolytic anemia as an irAE. Pem is available for renal cell carcinoma and UC; Funabashi *et al*. reported real‐life a clinical practice study that demonstrated that grade 3 or higher anemia occurred in 2.9% of patients with advanced UC refractory to cisplatin,^20^ with a short mean follow‐up of 7.7 months. Conversely, Williams *et al*. described a case of jaundice due to AIHA and cholangitis occurring two years after the completion of Pem treatment for melanoma. Therefore, greater attention should be paid to the development of hematologic toxicity following prolonged treatment with Pem.^15^

## Conclusion

We present the first case of UTUC with ES that was successfully treated using corticosteroids. ES should be considered in the differential diagnosis of patients with Pem‐induced hematologic toxicity.

## Author contributions

Shota Kakita: Writing – original draft; writing – review and editing. Tomohiro Matsuo: Writing – original draft; writing – review and editing. Masaharu Ohki: Writing – review and editing. Ayaka Tsuchiyama: Writing – review and editing. Takuji Yasuda: Writing – review and editing. Hiromi Nakanishi: Writing – review and editing. Kensuke Mitsunari: Writing – review and editing. Kojiro Ohba: Writing – review and editing. Ryoichi Imamura: Supervision; writing – review and editing.

## Conflict of interest

The authors declare no conflicts of interest.

## Approval of the research protocol by an Institutional Reviewer Board

No ethical approval was required for this case report.

## Informed consent

Written informed consent was obtained from the patient.

## Registry and the Registration No. of the study/trial

Not applicable.
